# Relationship between cerebellar structure and emotional memory in depression

**DOI:** 10.1002/brb3.738

**Published:** 2017-05-29

**Authors:** Li‐Yan Xu, Fang‐Cheng Xu, Can Liu, Yi‐Fu Ji, Jin‐Min Wu, Ying Wang, Hai‐Bao Wang, Yong‐Qiang Yu

**Affiliations:** ^1^ Department of Radiology The First Affiliated Hospital of Anhui Medical University Hefei China; ^2^ Department of Neurology The First Affiliated Hospital of Anhui Medical University Hefei China; ^3^ The Centre of Anhui Mental Health and The Fourth Hospital of Hefei Hefei China

**Keywords:** cerebellum, emotional memory, magnetic resonance imaging, major depressive disorder, voxel‐based morphometry

## Abstract

**Background:**

A few studies have been conducted on the relationship between cerebellar volume and emotional memory or clinical severity in major depressive disorder (MDD). In this study, we aimed to compare the volume and density of the cerebellar gray matter (GM) in patients with MDD and in healthy controls (HCs) and explore the association between these cerebellar parameters and measurements of emotional memory and clinical severity.

**Method:**

Voxel‐based morphometry (VBM) and Individual Brain Atlases using Statistical Parametric Mapping (IBASPM) were used to assess GM density and volume in the cerebellum, respectively, in patients with MDD and the HCs. Indicators of emotional memory performance were measured, including the hit rate (HR), rate of false alarm (FA), precision (Pr = HR − FA) and emotional memory enhancement [∆Pr = Pr(emotion) − Pr(neutral)] values. Beck Depression Inventory (BDI) scores were used to measure the severity of depression.

**Results:**

In the patients with MDD, the GM density was decreased in three cerebellar cortical regions and increased in three cerebellar cortical regions (*p* < .005). The GM volumes in eight cerebellar cortical regions were significantly smaller in the patients with MDD than in the HC subjects (*p* < .05). In the patients with MDD, the GM volume was correlated with the ∆Pr (*p* < .05) in two cerebellar cortical regions. The BDI scores were significantly correlated with the relative GM densities (*p* < .05) in 5 cerebellar cortical regions, and the GM volumes in 13 cerebellar cortical regions were correlated with the BDI scores in patients with MDD.

**Conclusions:**

Emotional memory and the severity of depressive symptoms are associated with structural changes in both the posterior and anterior GM regions in the cerebellum in patients with MDD. These findings could be useful for improving our understanding of the neurobiological mechanisms underlying emotional memory and explaining the abnormalities of the neural correlates that are associated with MDD.

## 1 INTRODUCTION

Damage to the posterior lobule of the cerebellum can lead to clinical cerebellar cognitive‐affective syndrome (CCAS; Schmahmann & Sherman, 1998). Patients with CCAS may display affective symptoms, including changes in affect and emotional lability, without a cerebellar motor syndrome (Schmahmann & Caplan, 2006). The affective disturbances in CCAS, which range from emotional blunting and depression to disinhibition and psychotic features, suggest that the posterior lobule of the cerebellum is involved in cognitive functioning and emotion. Collective evidence obtained from anatomical (Schmahmann & Pandya, 1989), transcranial magnetic stimulation (Schutter & Van, 2009) and functional MRI (fMRI; Schmahmann,^2010^; Stoodley, 2012) studies clearly indicates that the cerebellum performs other functions in addition to motor control and is involved in cognition and emotion. Furthermore, compared with healthy controls, individuals with cerebellar lesions have been reported to have impaired subjective experiences of pleasant emotions in response to positive stimuli while retaining a normal emotional response to fear stimuli (Turner et al., 2007).

Emotionally arousing events are easier to remember than neutral events because of their encoding and vivid recollection (Bradley, Greenwald, Petry, & Lang, 1992). This phenomenon is known as emotional enhancement of memory (EEM; Sommer, Gläscher, Moritz, & Büchel, 2008; Talmi, Anderson, Riggs, Caplan, & Moscovitch, 2008). The amygdala and hippocampus play an important role in processing emotional memory, and both regions are involved in memory formation, consolidation, or retrieval operations (Dolcos, LaBar, & Cabeza, 2004; Kensinger & Schacter, 2008; LaBar & Cabeza, 2006; McGaugh, 2004 and Wolf, 2009). Abnormalities in emotional memory have been reported in patients with major depressive disorder (MDD; Gilboa‐Schechtman, Erhard‐Weiss, & Jeczemien, 2002). Patients suffering from depression have been shown to always remember or process information that matches the status of their mood. Therefore, individuals that suffer from depression display a greater bias toward negative emotional memory and a lower bias toward positive emotional memory (Holt et al., 2016). Patients with depression have a negative emotion “memory bias” feature, which is consistent with the “mood‐congruent memory (MCM)” hypothesis (Gilligan & Bower, 1983). Structural and functional abnormalities in brain regions that are related to emotional memory, such as the amygdala (Hamilton, Siemer, & Gotlib, 2008; Townsend et al., 2010), the hippocampus (Ahdidan et al., 2011; Tahmasian et al., 2013), the prefrontal cortex (PFC; Bermpohl et al., 2009; Grieve, Korgaonkar, Koslow, Gordon, & Williams, 2013), the anterior cingulate cortex (ACC; Davey, Harrison, Yücel, & Allen, 2012; Yucel et al., 2008), and the thalamus (Caetano et al., 2001; Greicius et al., 2007), have been described in individuals with MDD. The cerebellum plays an important role in MDD according to recent imaging studies in which abnormal activity (Naismith et al., 2010), decreased regional homogeneity (ReHo; Guo et al., 2011; Liu et al., 2010), increased functional connectivity between the cerebellum and temporal poles (Liu et al., 2012), decreased functional connectivity between the cerebellum and the default‐mode network (DMN; Liu et al., 2012), and reduced cerebellar volume (Baldaçara, Borgio, Lacerda, & Jackowski, 2008; Yucel et al., 2013) were observed. A previous fMRI investigation consistently visualized the activation of the cerebellum as patients with depression viewed a negative emotional film (Beauregard et al., 1998). However, the specific relationship between the cerebellar structure and emotional memory in depression remains to be elucidated.

Among the few studies that have focused on the cerebellar cortical volume in individuals with MDD (Baldaçara et al., 2008; Grieve et al., 2013), most of the results have indicated atrophy in the gray matter (GM) and white matter (WM), particularly in the posterior lobules of the cerebellum (VI and VII). Lobule VII includes the vermis at the midline and the hemispheric portions of lobules VIIA (Crus I and Crus II) and VIIB. Recently, studies by Depping et al. (2016, 2017) have shown an increased GM volume in lobules VIIA and IX in individuals with MDD. Schutter, Koolschijn, Peper, and Crone (2012) demonstrated that the posterior lobules of the cerebellum are significantly involved in processes that are associated with cognitive and emotional functions. The anterior region of the cerebellum appears to be mainly engaged in motor and somatosensory tasks. However, Epstein et al. (2011) reported a lack of segregation of emotional processing from cognitive and sensorimotor functions in individuals with depression. We speculated that both the anterior and posterior lobules of the cerebellum play an important role in the pathophysiology of MDD.

We explored the abnormalities in the cerebellar cortical region density and volume in patients with MDD using voxel‐based morphometry (VBM) and Individual Brain Atlases using Statistical Parametric Mapping (IBASPM). The cortical density and volume reductions in both the posterior and anterior subregions of the cerebellum were expected. Additionally, we assessed the correlations between both the structures of the anterior and posterior subregions of the cerebellar cortex and the clinical severity and emotional memory in depression.

## 2 MATERIALS AND METHODS

### 2.1 Participants

Sixty‐two subjects with MDD (25 males and 37 females, aged 17–74 years) and 130 healthy controls (HCs; 61 males and 69 females, aged 20–78 years) were recruited (No significant differences were found between the two groups in terms of gender or age, *p* > .05; Table S1). All participants were Chinese‐speaking and right‐handed. All MDD patients were recruited from the inpatient and outpatient clinics of the Department of Psychiatry at the First Affiliated Hospital of Anhui Medical University or the Centre of Anhui Mental Health. The patients were diagnosed based on the Structured Clinical Interview in the DSM‐IV. None of the subjects in the HC group had a history of psychiatric illness. The exclusion criteria included a serious physical illness, another psychiatric diagnosis or alcohol/substance abuse, a severe infection or a recent surgery, previous participation in a clinical drug study, pregnant and lactating women, and color blindness. All MDD patients were experiencing their first episode, received no medication prior to the scanning, and underwent clinical psychiatric examinations, including an evaluation based on the 17‐item Hamilton Depression Rating Scale (HAMD‐17). Using the Beck Depression Inventory‐I (BDI‐I), both the MDD and HC groups were instructed to rate their moods during the week preceding the study. The inclusion criteria for the MDD group included an HAMD‐17 score >7 (Frank et al., 1991) or a BDI‐I score >4 (Beck, Ward, Mendelson, Mock, & Erbaugh, 1961). The BDI scores were used to assess the severity of depression (Mwangi, Matthews, & Steele, 2012). All participants provided written informed consent in accordance with the Institutional Review Board of Anhui Medical University, and all participants received monetary compensation for their time.

### 2.2 Behavior paradigm

In this study, we used 60 emotionally aversive and 60 neutral pictures that were selected from our own image set (Wang et al., 2010) and the International Affective Picture System (IAPS; Lang, Bradley, & Cuthbert, 2005). For each category, 30 pictures were used during the encoding phase (as the target items), and the 30 additional pictures were used during the recognition phase as novel stimuli (as foils). During the encoding phase, the subjects were required to determine the number of individuals in each image. The left mouse button was pressed by those who observed a “number of people ≤2”, while individuals who detected a “number of people ≥3” pressed the right mouse button. During the recognition phase, the participants were instructed to determine whether they had seen the picture before (was the picture presented in the first trial?) using the left or right mouse button as rapidly and accurately as possible.

The emotional arousal and valence levels of each picture were rated according to the Self‐Assessment Manikin (SAM) paradigm (nine‐point scales, 1 = sleepy or very unpleasant, 9 = very excited or very pleasant; Lang et al., 2005) by 20 additional volunteers (10 women and 10 men who did not participate in the experiment). The means and standard deviations (M (SD)) of the arousal and valence levels in response to the emotional and neutral pictures were as follows: 7.19 (0.52) and 2.40 (0.48); 2.76 (0.36) and 5.01 (0.16), respectively.

### 2.3 Scanning procedures

All imaging data were obtained using a General Electric Medical Systems HDxt 3.0T MRI scanner. A circular polarized head coil was used with foam padding to restrict head motion. High‐resolution T1‐weighted sagittal anatomical images were acquired using a 3D sequence [flip angle 15; repetition time/echo time 30/6.0 ms; matrix 256 × 256; field of view (FOV) 240 ×240 mm; 1.2 mm thickness; no gap; 166 slices; NEX = 1; image resolution 0.94 ×0.94 ×1.20 mm].

### 2.4 MRI data analysis

The structural T1‐weighted imaging data of the MDD patients and the HCs were processed using statistical parametric mapping software (SPM8, Wellcome Department of Cognitive Neurology, UK; http://www.fil.ion.ucl.ac.uk/spm; RRID:SCR_007037) on the Matrix Laboratory, MATLAB R2008a platform (MathWorks, Natick MA, USA). VBM (http://dbm.neuro.uni-jena.de/vbm) and IBASPM (http://www.thomaskoenig.ch/Lester/ibaspm.htm; RRID:SCR_014196) were performed for all subjects.

#### 2.4.1 Automated cerebellum density using VBM

The current study employed the VBM8 toolbox (http://dbm.neuro.uni-jena.de/vbm), which utilizes the unified segmentation approach, which is implemented in SPM8. The images were resampled at a resolution of 1 × 1 × 1 mm and normalized to the SPM standard template. Then, the bias field was corrected, and the images were automatically segmented. The structural T1‐weighted 3D images were segmented into GM, WM and cerebrospinal fluid (CSF). In addition, native space and DARTEL‐imported versions of the tissues were also generated. Subsequently, a custom template was created. DARTEL was applied to conduct the spatial normalization. This procedure was performed to achieve a more accurate inter‐subject registration of the images by registering the individual structural images to an asymmetric customized T1‐weighted template based on different samples rather than a standard T1‐weighted template. The resulting GM images were smoothed with an 8‐mm full‐width at half‐maximum (FWHM) Gaussian kernel, modulated, and spatially normalized using the Montreal Neurological Institute (MNI) template (http://www.mni.mcgill.ca/).

#### 2.4.2 Automated cerebellum volume using IBASPM

The postprocessing and image analysis data of both the MDD patients and the HCs were obtained using the IBASPM toolbox, which is implemented in MATLAB (http://www.thomaskoenig.ch/Lester/ibaspm.htm; RRID:SCR_007110). An SPM8 spatial normalization of the 3D T1‐W images was applied to the Montreal Neurological Institute (MNI) T1 template. After the normalization, the SPM segmentation function, which is included in the SPM8 toolbox, was implemented to segment the individual MRI images into the three types of tissues (GM, WM, and CSF). The normalization and segmentation procedures were similar to those that were used in the VBM procedure described above. During the automatic labeling stage, the individual GM voxels were labeled based on an anatomical atlas and included 116 cortical regions and the transformation matrix that was obtained during the normalization process. An individual brain atlas, which consisted of 116 different cortical regions, including 90 cerebral and 26 cerebellar cortical regions, was automatically created for each subject by the IBASPM toolbox. The cortical volume in each region of the cerebellum was corrected by the whole volume in all 116 regions.

### 2.5 Statistical analysis

The statistical analyses of the behavioral data were performed using the Statistical Package for the Social Sciences (SPSS; RRID:SCR_002865) version 13.0 software for Windows (SPSS, Chicago, IL, USA). We applied the Kolmogorov–Smirnov test and a normal plot to determine the normality of the distribution.

The behavioral indices included the mean reaction time and performance. The hit rate (HR), rate of false alarm (FA), precision (Pr = HR − FA) and emotional memory enhancement [∆Pr = Pr(emotion) − Pr(neutral)] values were acquired. Using the paired‐samples *t* test, the differences in the mean reaction time (including both accurate and false trials) and Pr for the negative emotion (E‐Pr) and neutral (N‐Pr) images between the MDD and HC groups were analyzed. An independent samples *t* test was performed to examine the differences in the ∆Pr and BDI scores between the two groups. A bivariate correlation with the Pearson correlation coefficient was used to assess the relationships among the E‐Pr, N‐Pr, ∆Pr and BDI scores.

The significance of the differences in the cerebellar cortical region density and volume between the MDD patients and the HCs was tested using an independent samples *t* test in a whole‐brain analysis using SPM (*p* < .05, uncorrected, cluster 50 voxels). Whole‐brain correlations were assessed between the ∆Pr, BDI scores and cortical density (*p* < .05, uncorrected, cluster 50 voxels). The counts of the supra‐threshold active voxels and labeling of the anatomical clusters were performed using MRIcro software with the template‐based automated anatomical labeling (AAL) tool (http://www.mccauslandcenter.sc.edu/mricro).

A two‐samples *t* test was performed to assess the differences in the volume values of individual subregions in the cerebellar cortex in the MDD and HC groups using SPSS (*p* < .05). Correlations between the relative cortical volume of individual subregions and the ∆Pr and BDI scores were analyzed (*p* < .05).

## 3 RESULTS

### 3.1 Behavior results

**Figure 1:**
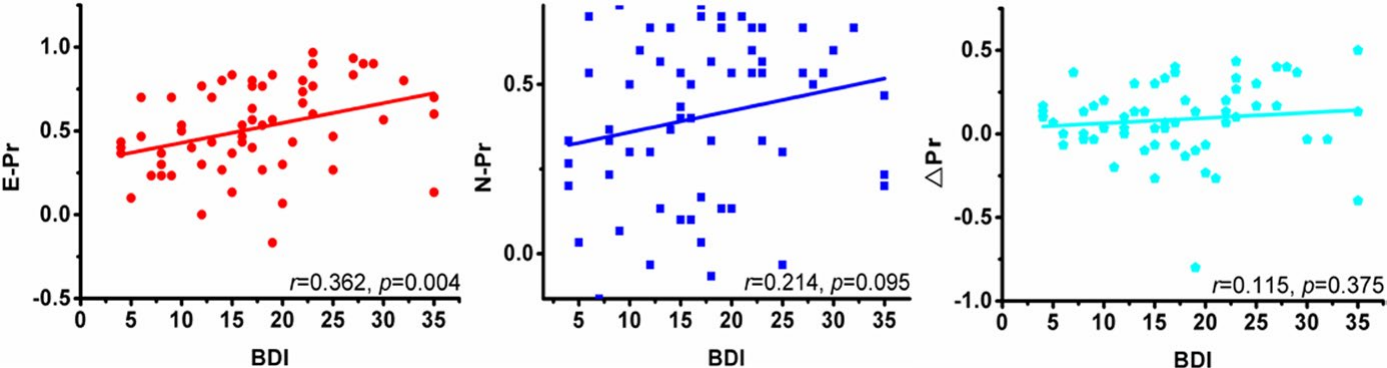
Correlation between behavior performance and BDI in MDD cases. E‐Pr was significantly positively correlated with BDI score (*p* < .05), while N‐Pr and ∆Pr had no significant correlation with BDI score (*p > *.05)

Compared with the negative emotion pictures, both the MDD and HC groups showed a higher mean reaction time and a lower mean accuracy rate in response to the neutral pictures (*p* < .05) as shown in Fig. S1. Moreover, the MDD patients showed a higher mean reaction time and a lower mean accuracy rate than the HC subjects in response to both the negative emotion pictures and the neutral pictures (*p* < .05). We observed no significant differences in the ∆Pr between the MDD and HC groups (*t* = 1.487, *p* > .05). A significant correlation was found between the BDI scores and the HAMD scores in patients with MDD as shown in Fig. S2. The BDI scores were significantly different between the two groups (*t* = −12.517, *p < *.001). In patients with MDD, E‐Pr was significantly positively correlated with the BDI score (*p < *.05), while N‐Pr and ∆Pr were not significantly correlated with the BDI score (*p *>* *.05; Figure 1).

### 3.2 Regional GM density differences between the groups

**Figure 2:**
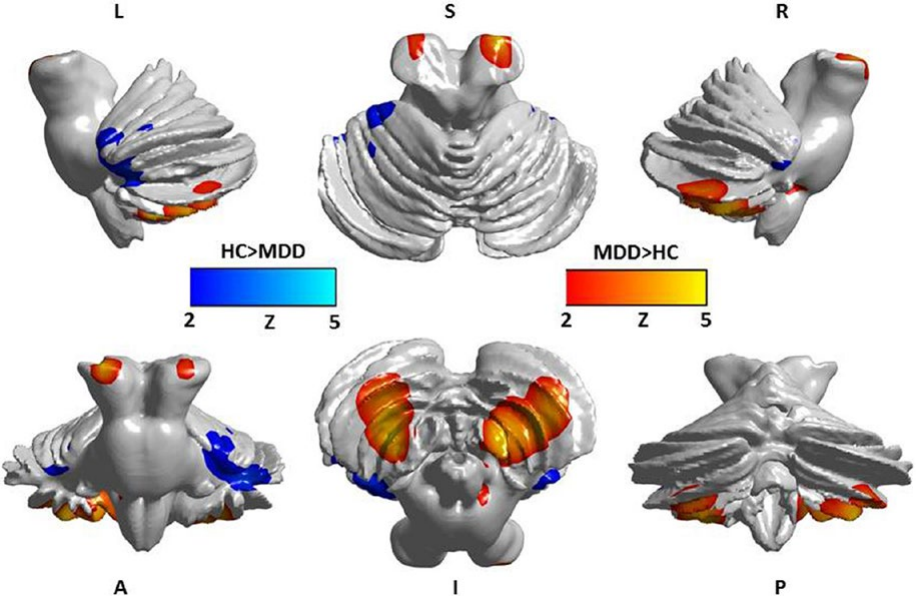
Significant differences between the two groups in gray matter density of cerebellar subregions. Cool color: decreased gray matter density in depression; Warm color: increased gray matter density in depression (*p* < .05)

A two‐samples *t* test revealed significant differences in the GM density of the cerebellum between the two groups. The patients in the MDD group had a decreased GM density in the left Cerebellum IV–V, left Cerebellum Crus I, and right Cerebellum VI. The GM density in the left Cerebellum VIII, right Cerebellum VIII and right Cerebellum IX was increased in the MDD patients (*p* < .05; Table 1, Figure 2).

**Table 1:** Significantly different subregions of the cerebellum in gray matter density between the two groups

Cerebellar Regions (AAL Labels)	MNI Coordinates			*Z*	*p*
	*x* (mm)	*y* (mm)	*z* (mm)		
MDD < HC					
Cerebelum_Crus1_L	−35	−43	−39	3.10	<.001
Cerebelum_4_5_L	−27	−38	−33	2.68	.004
Cerebelum_6_R	36	−39	−39	2.69	.004
MDD > HC					
Cerebelum_8_L	−23	−52	−52	4.93	<.001
Cerebelum_8_R	28	−57	−53	4.49	<.001
Cerebelum_9_R	17	−52	−47	4.50	<.001

*p* < .05, uncorrected.

### 3.3 Regional GM volume differences between the two groups

The GM volumes in eight cerebellar cortical regions, including the left Cerebellum IV–V, right Cerebellum IV–V, left Cerebellum VIIb, left Cerebellum VIII, right Cerebellum VIII, left Cerebellum X, right Cerebellum X and Vermis IV–V, were significantly smaller in the patients with MDD than in the HC subjects (*p* < .05). There were no cerebellar regions with increased GM volumes in the MDD patients (*p* < .05; Table 2).

**Table 2:** Significantly different subregions of the cerebellum in gray matter volume between the two groups

Cerebellar Regions (AAL Labels)	MNI Coordinates			*t*	*p*
	*x* (mm)	*y* (mm)	*z* (mm)		
MDD < HC					
Cerebelum_4_5_L	−14	−43	−17	2.953	.004
Cerebelum_4_5_R	18	−43	−18	2.509	.014
Cerebelum_7b_L	−31	−60	−45	3.002	.003
Cerebelum_8_L	−25	−55	−48	3.540	.001
Cerebelum_8_R	26	−56	−49	4.130	<.001
Cerebelum_10_L	−22	−34	−42	2.107	.036
Cerebelum_10_R	27	−34	−41	3.735	<.001
Vermis_4_5	2	−52	−6	2.473	.015

*p* < .05.

### 3.4 Correlation between GM densities in the cerebellum and emotional memory enhancement (∆Pr)

The GM densities in regions in the cerebellum were not correlated with the ∆Pr in the MDD patients (*p* < .05). Similarly, in the HC group, we observed no correlations between the GM densities in cerebellar regions and the ∆Pr (*p* < .05).

### 3.5 Correlation between GM volumes in the cerebellum and emotional memory enhancement (∆Pr)

In the MDD patient group, the GM volumes in two regions of the cerebellum, that is, the left Cerebellum VIIb and left Cerebellum IX, were negatively correlated with the ∆Pr (*p* < .05; Table 3). In the HC subjects, the GM volumes in the left Cerebellum IV–V, left Cerebellum VIIb, right Cerebellum VIIb and Vermis VII were negatively correlated with the ∆Pr (*p* < .05; Table 4).

**Table 3:** Significant correlation between GM volumes of subregions and the effect of emotional memory enhancement (∆Pr) in MDD

Cerebellar Regions (AAL Labels)	MNI Coordinates			*r*	*p*
	*x* (mm)	*y* (mm)	*z* (mm)		
Cerebelum_7b_L	−31	−60	−45	−.251	.049
Cerebelum_9_L	−10	−49	−46	−.257	.044

*p* < .05.

**Table 4:** Significant correlation between GM volumes of the cerebellum subregions and the effect of emotional memory enhancement (∆Pr) in HC

Cerebellar Regions (AAL Labels)	MNI Coordinates			*r*	*p*
	*x* (mm)	*y* (mm)	*z* (mm)		
Cerebelum_4_5_L	−14	−43	−17	−.195	.026
Cerebelum_7b_L	−31	−60	−45	−.216	.013
Cerebelum_7b_R	34	−63	−48	−.220	.012
Vermis_7	2	−72	−25	−.194	.028

*p* < .05.

### 3.6 Relationship between GM densities in the cerebellum and the severity of MDD (BDI scores)

**Figure 3:**
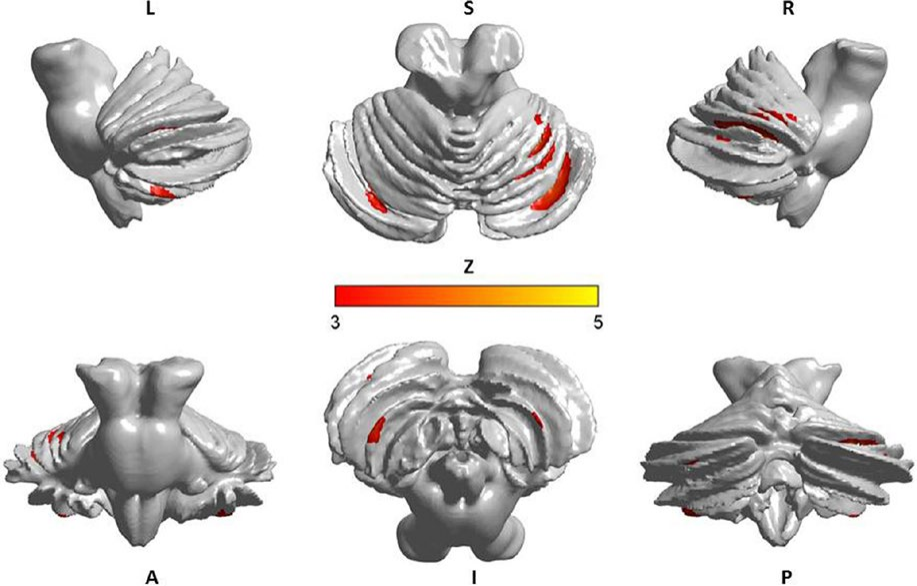
The relative GM densities in the left Cerebellum VI, right Cerebellum VI, left Cerebellum VIII, left Cerebellum Crus I and right Cerebellum Crus I were significantly correlated with BDI in the MDD patients (*p* < .05)

The relative GM densities in the left Cerebellum VI, right Cerebellum VI, left Cerebellum VIII, right Cerebellum VIII and left Cerebellum Crus I were significantly correlated with the BDI scores in the MDD patients (*p* < .05; Table 5, Figure 3).

**Table 5:** Significant correlation between GM densities of the cerebellum subregions and the severity of MDD (BDI scores)

Cerebellar Regions (AAL Labels)	MNI Coordinates			*z*	*p*
	*x* (mm)	*y* (mm)	*z* (mm)		
Cerebelum_Crus1_L	−30	−70	−31	3.37	<.001
Cerebelum_Crus1_R	31	−69	−30	3.64	<.001
Cerebelum_6_L	−31	−53	−31	3.76	<.001
Cerebelum_6_R	30	−52	−27	3.58	<.001
Cerebelum_8_L	−30	−56	−54	3.34	<.001

*p* < .05; uncorrected.

### 3.7 Relationship between GM volumes in the cerebellum and the severity of MDD (BDI scores)

The GM volumes in 13 cerebellar regions, including the left Cerebellum IV–V, right Cerebellum IV–V, left Cerebellum VI, right Cerebellum VI, left Cerebellum VIII, right Cerebellum VIII, left Cerebellum IX, right Cerebellum IX, left Cerebellum X, Vermis I–II, Vermis IV–V, Vermis VIII and Vermis IX, were significantly correlated with the BDI scores in the MDD patients (*p* < .05; Table 6).

**Table 6:** Significant correlation between GM volumes of the cerebellum subregions and the severity of MDD (BDI scores)

Cerebellar Regions (AAL Labels)	MNI Coordinates			*r*	*p*
	*x* (mm)	*y* (mm)	*z* (mm)		
Cerebelum_4_5_L	−14	−43	−17	−.339	.007
Cerebelum_4_5_R	18	−43	−18	−.315	.013
Cerebelum_6_L	−22	−59	−22	−.289	.023
Cerebelum_6_R	26	−58	−24	−.316	.012
Cerebelum_8_L	−25	−55	−48	−.309	.014
Cerebelum_8_R	26	−56	−49	−.300	.018
Cerebelum_9_L	−10	−49	−46	−.300	.018
Cerebelum_9_R	10	−49	−46	−.340	.007
Cerebelum_10_L	−22	−34	−42	−.362	.004
Vermis_1_2	2	−39	−20	−.273	.032
Vermis_4_5	2	−40	−11	−.372	.003
Vermis_8	2	−64	−34	−.284	.025
Vermis_9	2	−55	−35	−.450	.000

*p* < .05.

## 4 DISCUSSION

Currently, the role of the cerebellum in cognitive and affective processing is widely recognized. The circuits that connect the cerebellum to the cerebral cortices and paralimbic regions have been identified (Schmahmann, Weilburg, & Sherman, 2007; Stoodley & Schmahmann, 2009). However, cerebellar structural changes in MDD patients have been demonstrated. Patients with MDD exhibit atrophy in the GM and WM, particularly in the posterior lobules of the cerebellum (VI and VII; Baldaçara et al., 2008; Grieve et al., 2013). Recently, a study by Malte revealed (Depping et al., 2016, 2017) that patients with an acute MDD episode have a higher volume in Cerebellar lobule IX, Cerebellar lobule VIII and Cerebellar lobule VIIb than HCs. However, research studies regarding the changes in the cerebellar volume and emotional memory in patients with depression remain scarce.

In the present study, we investigated emotional memory and the cerebellar structural changes in patients with MDD. Compared with the HC subjects, the patients with depression displayed a significant impairment in emotional memory and a decreased volume in both the anterior and posterior lobules of the cerebellum. A regional analysis of the cortical density revealed a complex pattern of density abnormalities in patients with depression that involved a combination of increases and decreases in the cortical regions. In the present study, we found that the reductions in GM volume (but not in density) were associated with decreased emotional memory, while the reductions in GM density and volume were associated with the severity of depressive symptoms.

The results of our experiments demonstrated that individuals with MDD have higher mean reaction times and lower accuracy rates in response to both neutral and emotionally aversive images than HCs. Our data indicated that although emotional memory performance is reduced in MDD patients, the emotional memory enhancement effect is unchanged. The decreased accuracy in the recognition of negative and neutral images may reflect a general degradation in performance that is manifested in many cognitive tasks (Williams, Watts, MacLeod, & Mathews, 1988). The increased reaction times might be explained by the fact that patients with depression show a reduced performance in many types of cognitive tasks (Williams et al., 1988). Furthermore, the BDI scores were significantly correlated with the accuracy of the negative emotional picture recognition but not with the accuracy of the neutral picture recognition and the emotional memory enhancement. In the present study, we found that compared with the responses to the neutral images, the accuracy of the negative emotion image recognition was increased as the severity of depression increased. Although no significant correlations were observed between the N‐Pr and BDI scores, the *p*‐value was .095. A potential reason for the nonsignificant association between emotional memory enhancement and the BDI scores is that emotional memory enhancement was influenced by both E‐Pr and N‐Pr in our sample.

In the present study, the MDD patients exhibited structural changes not only in the posterior cerebellum but also in the anterior cerebellum and the flocculonodular lobe. Our results indicated that a decrease in the GM density in cerebellar lobules VI and VII was a marker of depression. Consistently, most findings indicated that bilateral regions in the posterior region of the cerebellum, including lobules VI and VII (Crus I, Crus II and Lobule VIIb), were engaged in executive functions and emotional processing (Schmahmann et al., 2007; Stoodley, 2012). Prior studies (Krienen & Buckner, 2009; O'Reilly, Beckmann, Tomassini, Ramnani, & Johansen‐Berg, 2010) have demonstrated that the activity in the prefrontal, posterior parietal and superior and middle temporal association areas as well as the cingulate gyrus and retrosplenial cortex were correlated with the activity in cerebellar lobules VI and VII. The lateral hemisphere of the cerebellum is functionally connected to the dorsolateral prefrontal cortex (dlPFC), suggesting that cerebellar lobules VI and VII are potentially involved in executive functioning (Habas et al., 2009). Furthermore, Crus I was functionally associated with the medial prefrontal and anterior cingulate cortices, supporting its involvement in default‐mode activity and emotional processing (Krienen & Buckner, 2009). These results indicate a functional connectivity between cerebellar lobules VI and VII and the cerebrum. These findings suggest that the decrease in the GM density in cerebellar lobules VI and VII may reflect altered cerebellar‐cerebrum connectivity patterns in MDD patients.

Our results also revealed structural changes in the posterior cerebellum (lobules VIII and IX), anterior cerebellum (lobules IV and V) and flocculonodular lobe (lobule X). A meta‐analysis has shown that motor tasks are localized in anterior lobes IV and V with a secondary representation in lobules VIIIa/b. Somatosensory tasks also involve the anterior lobe of Cerebellum lobules IV and V, with a secondary representation in lobule VIIIb. Stoodley (Stoodley & Schmahmann, 2010) proposed that there is a dichotomy between “sensorimotor” and “cognitive” processes, which was supported by clinical findings in cerebellar stroke patients. Using fMRI, Epstein et al. (2011) reported a lack of segregation of emotional processing and cognitive and sensorimotor functions in patients with depression. The present study also showed a volume decrease in the flocculonodular lobe in the MDD patients. The flocculonodular lobe consists of the nodule and the flocculus. The flocculonodular lobe mainly functions as a regulator of the vestibular system and an adjustor in response to vestibular damage. A previous study (Soza & Aviles, 2007) has found that emotion processing systems likely influence the vestibular system. Soza and Aviles found that patients with vestibular vertigo syndromes exhibit high depressive symptoms. However, patients with depression might exhibit dizziness symptoms. Therefore, we have reason to believe that the flocculonodular lobe is involved in MDD. Future research should elaborate on our findings regarding the increased GM density in cerebellar lobules VIII and IX. Typically, an increased GM volume signifies an ineffective functional state in the respective brain system (Romanczuk‐Seiferth et al., 2014). We hypothesized that the function of these two cerebellar regions was abnormal in MDD patients. Taken together, these data suggest that widespread GM structural abnormalities in the cerebellum are present in MDD patients. Not only were the posterior regions of the cerebellum involved in depression but the anterior regions and flocculonodular lobe of the cerebellum were also involved.

Additionally, the reductions in the GM volume (but not in the density) in the cerebellum were associated with a decreased emotional memory, and the reductions in GM density and volume were associated with the severity of depressive symptoms. The decrease in GM volume was not always accompanied by a corresponding reduction in GM density. Recently, a decrease in the hippocampal volume was shown to be correlated with an increase in the neuronal density in depressive monkeys (Willard, Riddle, Forbes, & Shively, 2013). The reduction in the volume was attributed to changes in the glial number and the extent of neuropil aggregation (Willard et al., 2013). A postmortem study by Cobb et al. (2013) showed that the CA1 pyramidal neuron density increased with the duration of the illness and recurrent/chronic major depression. In the present study, we observed that the GM volumes in two regions in the cerebellum were involved in emotional memory processing in depression. Lobule VII in the cerebellum is engaged in emotional memory in normal conditions. Compared with the healthy control subjects, the patients with depression displayed altered regions in the cerebellum, such as in left lobule VII, which was engaged in emotional memory processing in the MDD patients. Lobule IX has been hypothesized to act as a part of the DMN (Habas et al., 2009; Krienen & Buckner, 2009). The activation of the DMN is associated with memory retrieval and emotional memory processing (Xu et al., 2014).

In addition, we found that the reductions in GM density and volume were associated with the severity of depressive symptoms. Several neuroimaging studies (Dubin et al., 2012; Gaffrey et al., 2011) have shown a relationship between depression severity and functional and structural abnormalities in the cerebrum. However, limited previous studies have focused on this association in the cerebellum. Based on the current findings, we propose that both the posterior and anterior regions of the cerebellum are associated with the severity of depression, and both regions play an important role in the pathophysiology of MDD.

## 5 CONCLUSION

Although emotional memory performance is reduced in depression, the emotional memory enhancement effect still exists. Both the posterior and anterior regions of the cerebellum are involved in depression. Additionally, the reductions in GM volume (but not in the density) are associated with decreased emotional memory, while the reductions in GM density and volume are associated with the severity of depressive symptoms.

## CONFLICT OF INTERESTS

The authors declare that there is no conflict of interest.
