# Supplementary figures and images for: Relationship between cerebellar structure and emotional memory in depression
Source: Brain Behav. 2017 May 29;7(7):e00738. doi: 10.1002/brb3.738 (PMC5516611; doi:10.1002/brb3.738)

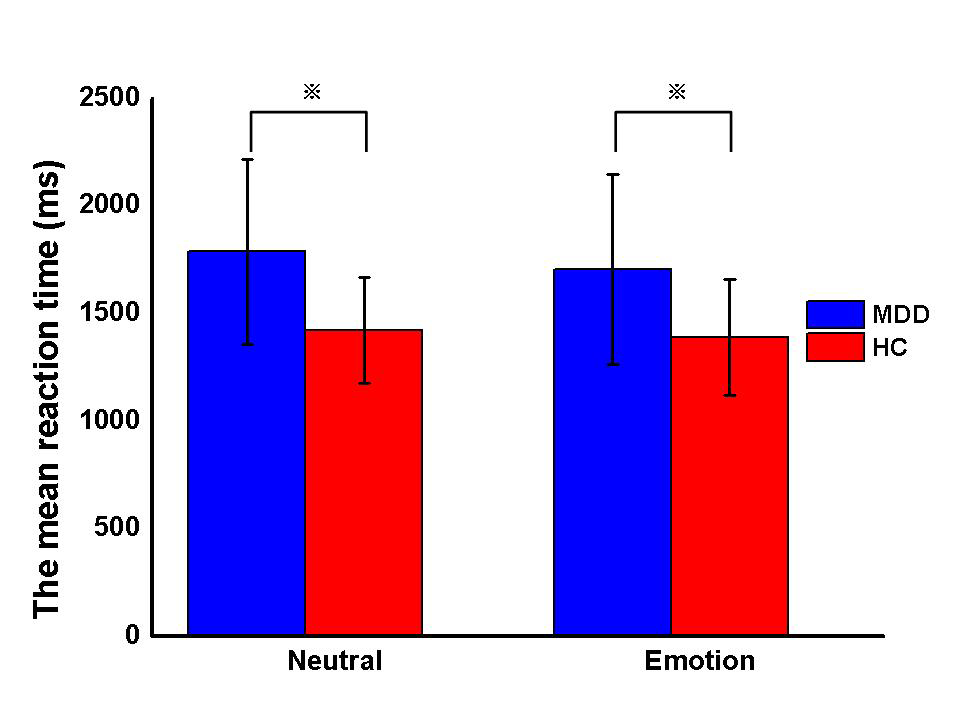

Supplement: Supplementary file 1 [file BRB3-7-e00738-s001.tif]

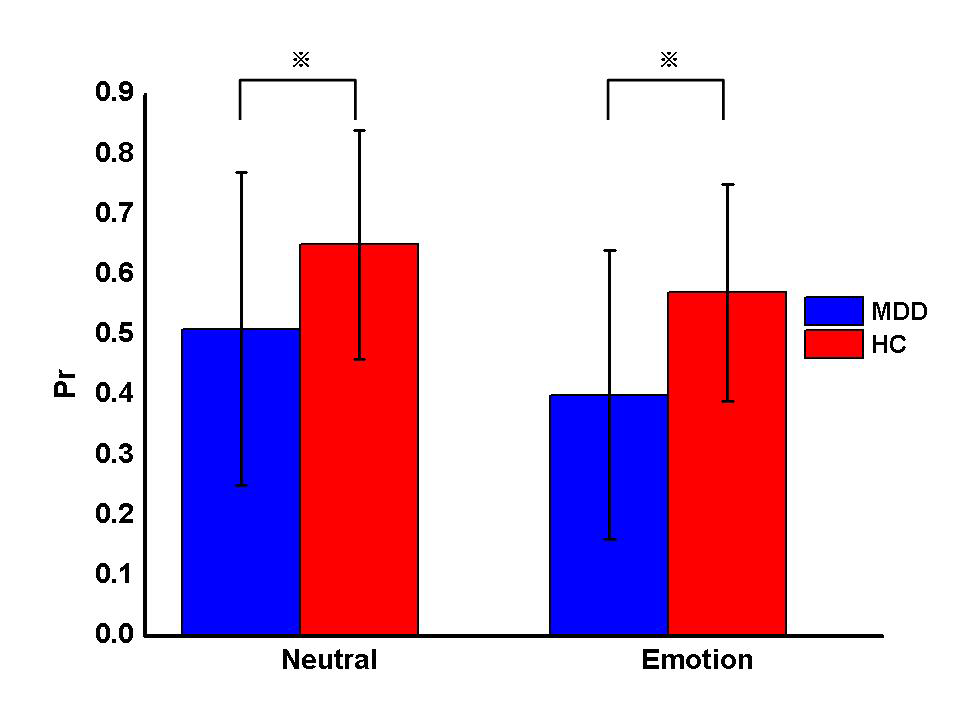

Supplement: Supplementary file 2 [file BRB3-7-e00738-s002.tif]

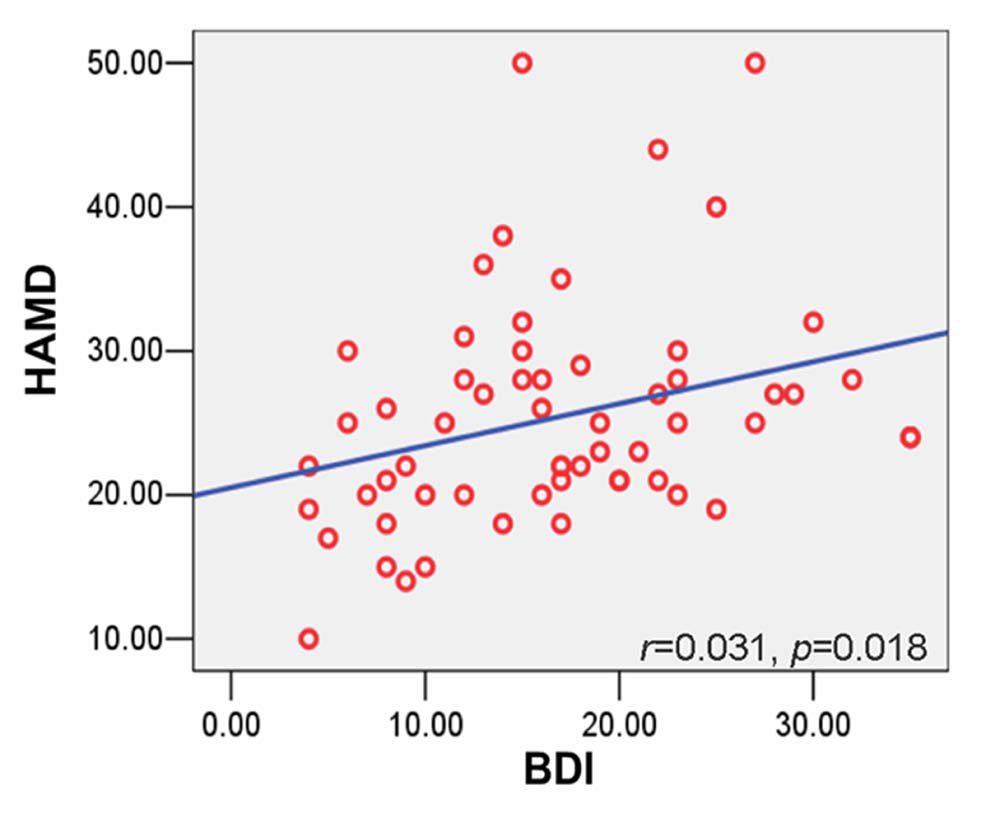

Supplement: Supplementary file 3 [file BRB3-7-e00738-s003.tif]
