# Supplementary material for: Relationship between cerebellar structure and emotional memory in depression
Source: Brain Behav. 2017 May 29;7(7):e00738. doi: 10.1002/brb3.738 (PMC5516611; doi:10.1002/brb3.738)
Supplement: Supplementary file 4 [file BRB3-7-e00738-s004.doc]

Supplementary Table 1. Demographic and Clinical Details

| Measures | HC  (n = 62) | MDD  (n = 134) | t/X^2^ | p |
| --- | --- | --- | --- | --- |
| Demographic/clinical measures |  |  |  |  |
| Age (years) | 42.68±16.94 | 47.84±17.31 | 1.961 | 0.051 |
| Gender | 61M;69F | 25M;37F | 0.800 | 0.800 |
| Education (years) | 11.50±2.91 | 10.0±3.76 | -1.384 | 0.071 |
| HRSD_17_ | - | 26.33±6.96 | - | - |
| BDI-Ⅰ | 2.65±2.59 | 19.58±6.84 | 12.52 | < 0.0001 |

Abbreviations: HRSD_17_, 17-item Hamilton Rating Scale for Depression; BDI, Beck Depression Inventory; HC, Health control; MDD, Major Depressive Disorder.

**(Note: P<0.05)**

For Review Only Fig.1 Correlation between behavior performance and BDI In MDD cases, E-Pr was significantly positively correlated with BDI score (p ＜0.05), while N-Pr and △Pr had no significant correlation with BDI score (p ＞0.05)

Fig.2 significantly different subregions of the cerebellum in gray matter density between the two groups Cool color: decreased gray matter density in depression; Warm color: increased gray matter density in depression

Fig. 3 Statistical maps showing subregions between the BDI score and relative GM densities right Cerebelum Ⅷ, left Cerebelum Ⅵ, left Cerebelum Ⅷ and left Cerebelum Crus1 were significantly correlated of the VBM maps in depression

Supplementary Fig.1 Difference of the mean reaction time (A) and accuracy rate (B) reacted to emotional (E) and neutral pictures (N) in MDD and HC.

Supplementary Fig.2 Significant correlation between BDI and HAMD sores in MDD.
